# Guidance or Misdirection? Unpacking the Role of Feedback in Health Preference Assessments

**DOI:** 10.1002/hec.70093

**Published:** 2026-03-03

**Authors:** Mesfin G. Genie, Shelby D. Reed, Semra Ozdemir

**Affiliations:** ^1^ Newcastle Business School, College of Human and Social Futures The University of Newcastle Newcastle NSW Australia; ^2^ Department of Health Outcomes Research and Policy Auburn University Auburn Alabama USA; ^3^ Department of Population Health Sciences Duke University School of Medicine Durham North Carolina USA; ^4^ Duke University School of Medicine Duke Clinical Research Institute Durham North Carolina USA

**Keywords:** choice consistency, discrete choice experiment, feedback intervention, medical devices

## Abstract

This study investigated the impact of providing feedback to respondents on a dominance‐structured choice task on subsequent choice behavior in a discrete choice experiment (DCE). The DCE was conducted among 626 patients with heart failure. Respondents were given a dominance‐structured choice task in which two devices (Device A and Device B) offered no benefits but carried risks compared to a “No Device” option. Among those who selected a device option (*N* = 340), half received feedback and an opportunity to revise their choice, while the other half did not. The effect of feedback on preference for the “No Device” option and choice consistency was examined using multinomial, heteroscedastic multinomial logit, and heteroscedastic latent‐class logit models. Among those who received feedback (*N* = 170), 71% continued to choose the device options. Feedback recipients were more likely to choose the “No Device” option in subsequent questions (*p* < 0.01). Feedback led to a 25% reduction in choice consistency (*p* < 0.01) and an increased likelihood of choosing the “No Device” option. Impact on consistency varied across latent classes: feedback decreased consistency in the risk‐sensitive class but increased consistency in the anti‐device class, highlighting potential unintended consequences. Further research is needed to understand its effects in different contexts and samples.

## 1 Introduction

Discrete choice experiments (DCEs) are widely used in applied economics for eliciting preferences over multidimensional alternatives. DCEs present respondents with hypothetical yet clinically relevant or policy‐relevant scenarios, allowing analysts to recover marginal utilities, quantify trade‐offs, and estimate willingness‐to‐pay (WTP) measures (Holmes et al. 2019; Lancsar and Louviere 2008b; Soekhai et al. 2019). The approach has become a widely used method in health economics for evaluating healthcare services and technologies and quantifying treatment benefit‐risk trade‐offs (Clark et al. 2014; De Bekker‐Grob et al. 2012; Johnson and Zhou 2016; Tervonen et al. 2023; Whichello et al. 2023). To support valid inference, DCEs typically incorporate tutorials, comprehension checks, and practice tasks to familiarize respondents with the decision environment and mitigate measurement error (Janssen et al. 2018; A. Pearce et al. 2021; A. M. Pearce et al. 2020; Vass et al. 2020).

However, a growing body of methodological research demonstrates that such “guidance” features are not always neutral. Advance disclosure of task structure, instructional choice sets (ICS), or “time‐to‐think” provisions have all been shown to influence not only scale (error variance) but also systematic preference parameters. ICS, for example, are intended to train respondents to make trade‐offs, but can anchor respondents on early attribute levels, thereby shifting WTP estimates (Meyerhoff and Glenk 2015). In food safety DCEs, advance disclosure and ICS generated detectable learning effects and altered status quo tendencies (Abate et al. 2018). Allowing respondents extra deliberation time increased choice consistency but also shifted attribute weights and class membership for latent preference groups (Veldwijk et al. 2020). Even interventions designed to reduce hypothetical bias, such as cheap talk, consequentiality scripts, or opt‐out reminders, have been found to alter marginal rates of substitution (Alemu and Olsen 2018; Cummings and Taylor 1999; Kassie et al. 2022; Özdemir et al. 2009). Overall, these findings suggest that feedback or guidance elements intended to improve data quality can shape preferences.

Within this landscape, dominated‐choice validity checks represent a particularly under‐examined intervention. A dominated alternative, one that is strictly worse on all attributes, is expected to be universally rejected if respondents understand the task. In practice, however, 10%–20% still select dominated options (Johnson et al. 2019; Veldwijk et al. 2023), raising concerns about comprehension, heuristics, label effects, or latent preferences for action over inaction (Dolan et al. 2015). Currently, such tasks are treated as ex‐post diagnostic screens, with failures used to flag inattentiveness or justify exclusions (Jonker et al. 2022; Tervonen et al. 2018). Methodological guidance cautions against this practice because dominated‐choice tasks have poor sensitivity and specificity, but more importantly, dominance failures are almost never disclosed to respondents in real time. Thus, the potential of dominance checks as feedback interventions remains unexplored.

Normative rational‐choice theory treats dominance as a main requirement of coherent preferences: if one option is strictly worse on all relevant outcomes, a rational decision‐maker should never choose it. In their classic analysis of rational choice and framing, Tversky and Kahneman identify invariance and dominance as normatively essential axioms of choice and show how violations often arise when equivalent problems are framed differently, rather than because individuals lack preferences (Tversky and Kahneman 1986). In our study, the “dominated” device is defined relative to the attribute space presented to respondents and the clinical context of heart failure; within that frame, it is strictly worse on all benefit and risk outcomes, and so functions as a clean test of whether respondents recognize the dominance relation implied by the attributes we provide.

At the same time, a long‐standing behavioral tradition cautions against interpreting all dominance violations as irrational or meaningless. Simon's work on bounded rationality emphasizes that real decision makers have limited cognitive resources and therefore rely on simplifying procedures or satisficing rather than global optimization (Simon 1955). Fast‐and‐frugal heuristics, such as “one‐reason” decision rules, can ignore much of the available information and still perform well in many environments (Gigerenzer and Goldstein 1996). Similarly, the adaptive decision‐maker framework shows that people switch between strategies that trade off effort and accuracy depending on task complexity and time pressure (Payne et al. 1993). In health‐related DCEs, respondents have been shown to employ lexicographic or noncompensatory rules, to attend selectively to attributes, or to use elimination‐by‐aspects strategies, all of which can generate choices that appear dominated from the analyst's perspective while still reflecting structured preferences (Erdem et al. 2014; A. Pearce et al. 2021; Ryan and Bate 2001; Scott 2002). Our use of a dominance‐structured task is therefore grounded in the rational‐choice view of dominance as a benchmark of coherence, but interpreted against this broader behavioral evidence that some “dominance failures” may reflect alternative decision strategies rather than simple errors.

Theoretical and empirical work from outside the DCE literature suggests that such feedback could matter. Feedback intervention theory (FIT) (Kluger and DeNisi 1996) conceptualizes feedback as the provision of information regarding one's performance on a given task that can direct attention, increase effort, correct cognitive biases, and reduce error variance. According to FIT, feedback that focuses participants on task performance can signal the need for more effort (Kluger and DeNisi 1996). More broadly, feedback can serve as an informational input that may help decision‐makers update beliefs and adjust subsequent choices, while cognitive psychology highlights its role in clarifying task structure and signaling mistakes, thereby improving decision accuracy (Shute 2008; Hattie and Timperley 2007). Across domains, feedback has been shown to shift subsequent behavior. In education, interim or relative performance information can boost subsequent test scores by providing both information and motivation, with effects strongest among higher‐ability or less‐informed students (Azmat and Iriberri 2010; Bandiera et al. 2015). In the workplace, feedback to frontline public‐sector employees has been shown to increase productivity; for example, daily organ‐donor registrations rose by 25% when call‐center agents received performance information (House et al. 2022). In consumer markets, cost‐related feedback has been shown to increase consumer knowledge and change consumption behavior (Hutton et al. 1986). In environmental economics, better‐informed respondents displayed a higher scale (lower variance) than less well‐informed respondents (LaRiviere et al. 2014). This cross‐disciplinary evidence suggests that feedback interventions can alter preferences and choice behavior as well as consistency, with effects distributed unevenly across agents.

Our study leverages this insight by embedding a dominance‐structured training task within the DCE and randomizing whether respondents who selected a device with no benefits but with associated risks received immediate feedback. We treat this experiment not as a diagnostic test but as an opportunity to assess whether feedback improves performance. Specifically, we evaluate two hypotheses:

Hypothesis 1 (Consistency): Providing feedback to respondents is expected to increase choice consistency in subsequent tasks, reflected in higher scale parameter (lower error variance).

Hypothesis 2 **(**Behavioral adjustment): Providing feedback is expected to reduce the likelihood that respondents select a device in subsequent tasks, nudging them toward the opt‐out (“No Device”) alternative.

By testing these hypotheses, we aim to assess whether real‐time feedback can enhance the internal validity of DCE data and conceptualize the boundary between information provision and behavioral influence in stated‐preference research. Accordingly, the main contribution of this paper is methodological rather than substantive. We use a single, well‐characterized benefit‐risk DCE in the context of heart failure devices to examine how an explicit feedback prompt embedded in a dominance‐structured training task affects choice behavior. Methodological work in health DCEs frequently builds on existing datasets to study design features or modeling innovations (Hauber et al. 2016; Vass et al. 2018), and our analysis follows this tradition by using an already completed DCE to investigate the role of a dominance‐structured task and feedback in shaping choice behavior. The intended audience is researchers designing and analyzing stated‐preference studies, particularly where guidance or feedback elements are incorporated into survey instruments. Our analysis should not be interpreted as a challenge to the validity of stated‐preference methods as a whole. Consensus‐based good‐practice guidelines, including the ISPOR Task Force reports, document that carefully designed DCEs offer reliable and policy‐relevant evidence in healthcare (Bridges et al. 2011; Hauber et al. 2016). Moreover, evidence shows that well‐designed DCEs can provide policy‐relevant estimates and reasonable predictions of health‐related behaviors (Bridges et al. 2011; De Bekker‐Grob et al., 2012; Lancsar and Louviere 2008a; Merlo et al. 2020; Ozdemir et al. 2024; Quaife et al. 2018; Soekhai et al., 2019). Our findings therefore focus on the consequences of a specific design choice, feedback during a dominance‐structured task, and not on the broader legitimacy of stated‐preference evidence.

The remainder of this paper is organized as follows: Section 2 presents the study design and experimental manipulation. Section 3 discusses the empirical strategy, followed by the results in Section 4. Section 5 discusses the findings and implications. Section 6 provides concluding remarks.

## 2 Study Design

### 2.1 Context

This study is part of a larger stated‐preference study that focuses on assessing heart failure patients' acceptance of therapeutic risks in exchange for improved effectiveness with implantable devices. In a web‐based DCE survey, a series of choice questions asked respondents to choose among two device options and a “No Device” option. The options were defined by the following attributes: 1) An additional year of physical functioning equivalent to New York Heart Association (NYHA) class III or a year with improved (i.e., class II) symptoms, or both; 2) 30‐day mortality risks ranging from 0% to 15%; 3) in‐hospital complication risks ranging from 0% to 40%, and 4) a remote adjustment device feature. The no‐device option was defined by a health trajectory representing 3 years with physical functioning and symptoms equivalent to NYHA class III, followed by 2 years with functioning and symptoms equivalent to NYHA class IV. Table 1 presents the list of attributes and levels, with further study details reported elsewhere (Reed et al. 2022). This study uses data from the experiment reported in Reed et al. (2022) but addresses a distinct question. While Reed et al. (2022) focused on the main preference results, reporting the substantive benefit‐risk trade‐offs for heart failure devices, here we exploit the experiment's two‐arm design (with vs. without feedback on dominance‐structured choices) to investigate how such feedback influences respondents' choice behavior. In the original fieldwork, respondents who selected one of the device options in the dominance‐structured task were randomly allocated to one of two conditions. This randomization was pre‐specified as part of the survey design, but the existence of these two arms was not reported in Reed et al. (2022). The present study addresses this omission by describing the feedback and no‐feedback arms in full and by examining their consequences for choice behavior.

**TABLE 1:** Study attributes and levels.

Attributes	Levels			
Benefits				
Disease trajectory Years in NYHA class II, III and IV representing potential: Improvements in functioning Extended duration before worse functioning Longer survival duration		Years in		
		NYHA II	NYHA III	NYHA IV
	No‐device scenario	0	3	2
	1‐year gain in NYHA II	1	3	2
	1‐year gain in NYHA III	0	4	2
	1‐year gains in NYHA II and III	1	4	2
Device‐associated risks				
Mortality risk All‐cause death within 30 days of device implantation	0%^a^ 2% 5% 10% or 15%			
Complications risk An adverse event that would require a 2‐day hospital stay with no long‐term health consequences	0%^a^ 5% 15% 40%			
Device features				
Remote device programming Device that can or cannot be adjusted via internet connection	No^a^ Yes			

^a^ This level also represents the no‐device scenario.

**FIGURE 1:**
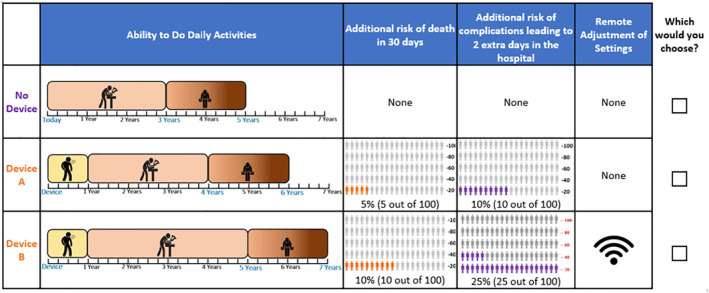
Example choice task.

The D‐efficient design was generated using SAS, version 9.4 (SAS Institute Inc. Cary, NC), and comprised 40 blocks of 8 experimentally controlled choice questions (see Figure 1 for an example choice task). Each participant was randomly assigned to answer one of the blocks. Participants were recruited from two sources (detailed in Reed et al. 2022): a national web panel (Kantar Health) of individuals in the United States who self‐reported a diagnosis of heart failure, and a cohort of patients with a physician‐verified diagnosis of heart failure treated at Duke University Health System (DUHS). We accounted for this in our analysis, and tests of differences by recruitment source are reported in the Supporting Information S1. Individuals younger than 18 years of age or those with congenital heart disease were excluded. The study protocol was reviewed and approved by the DUHS Institutional Review Board.

### 2.2 Experimental Manipulation: The Feedback Mechanism

The study was designed to simulate a scenario in which participants with heart failure faced the prospect of surviving three more years with physical functioning equivalent to NYHA class III and 2 years with class IV functioning, followed by death. In experimentally designed choice questions, they were presented with a choice between two device options, with the anticipation that these devices would offer some benefits, or the option of not using a device. The no‐device option was constant across all choice questions.

**FIGURE 2:**
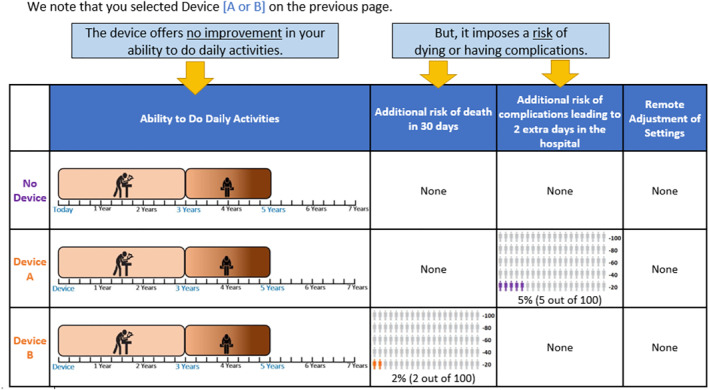
Dominated choice question and feedback intervention.

As part of the training materials, a dominance‐structured choice task was shown in which the two device options offered no benefits but were associated with increased risks, with the expectation that individuals would choose the no‐device option (see Figure 2). Following the addition of the dominance‐structured training task to the instrument, we conducted six cognitive pretest interviews. In three cases, participants initially selected one of the device options in the dominance‐structured task. When this was probed, participants indicated they had made a mistake and stated they would switch to “no‐device”. Given that our target population was older adults and to minimize unintentional selection of the seemingly inferior option, we embedded a feedback experiment in the main survey (i.e., to determine whether pointing out the additional risks of the device options without any offsetting benefits would potentially be helpful). Participants who chose one of the device options (Device A or Device B) in this question were randomly assigned to one of two groups. The first group‐hereafter referred to as the *NOFEEDBACK* group, proceeded with the survey without receiving any feedback. The second group‐hereafter referred to as the *FEEDBACK* group, encountered a follow‐up screen^1^ explicitly stating, *“The device offers no improvement in your ability to do daily activities. But it imposes a risk of dying or having complications”* (see Figure 2). After receiving this feedback, they were given the opportunity to answer the same question again. This experimental setup allowed us to assess the impact of providing feedback on subsequent choice behavior. This dominance‐structured choice task and feedback intervention were intentionally included to serve both as a comprehension probe and as the basis for the feedback experiment. Although some participants during pretesting also selected a device option with no benefits but risks, the task was retained in the final design to allow systematic evaluation of feedback effects. This study differs from the original study by evaluating the effect of the feedback intervention.

## 3 Analysis

Responses to DCE tasks are modeled within the random utility maximization (RUM) framework (McFadden 1974). This assumes that the utility (*U*) of the heart failure device (*j*) faced by the respondent (*n*) in a choice task (*t*) depends on a systematic component (*V*) and an unobservable stochastic component (ε):

(1) $$U_{ntj}=V_{ntj}+\varepsilon_{ntj}$$

The systematic component of utility is a function of the attributes of the heart failure devices (Table 1) $x_{njt}=\left(x_{njt1},x_{njt2},\text{…},x_{njtK}\right)$, characteristics of respondents $z_{n}$, and a variable representing whether respondent n received feedback $F_{n}$, such that:

(2) $$V_{ntj}=f\left(x_{ntj},z_{n},F_{n},\beta_{n}\right)$$

the vector $\beta_{n}$ represent the preferences of the respondent (*n*) for the attributes (*k*).

We included a single dominance‐structured training task in which respondents chose between two device alternatives offering no health benefit but entailing additional risks and a “No device” option. Our analysis was limited to participants who chose a device option in this task, thus representing a non‐random sample of the study participants. We modeled the data from the FEEDBACK and NOFEEDBACK groups, separately, using multinomial logit (MNL) regressions to investigate the theoretical validity. We then investigated the impact of feedback provision on choice behavior in two ways. First, we investigated whether providing feedback created an unintended signal toward choosing the “no‐device” option. We first created a binary variable “feedback” which takes a value of one if the experiment included feedback and zero otherwise. We explored the impact of providing feedback on preference weights for the “no‐device” parameter by interacting the “no‐device” parameter with the feedback dummy variable using an MNL regression based on the model specification described in Equation (3).

(3) $$V_{ntj}=\left(\alpha +\alpha_{\mathrm{feedback}}F_{n}\right)1_{\left\{j=\mathrm{No}\;\mathrm{Device}\right\}}+\sum_{x}\beta_{x}X_{ntj}+\varepsilon_{ntj},$$

We use $1_{\left\{j=\mathrm{No}\;\mathrm{Device}\right\}}$ as an indicator that alternative j is the opt‐out option (“No Device”). This term essentially means the utility of the “No Device” option has a base intercept α plus an additional term $\alpha_{\mathrm{feedback}}$ if the respondent received feedback. Here, $F_{n}$ is a dummy variable equal to 1 if respondent n was assigned to the feedback group, and 0 otherwise. Thus, $\alpha_{\mathrm{feedback}}$ captures the additive effect of the feedback intervention on the utility of the opt‐out (“No Device”) option.

Because the design was labeled, we expected that labels could contribute to utility beyond the presented attributes. To account for this, we estimated alternative‐specific conditional logit (ASCL) models with alternative‐specific constants (ASCs) to capture label utility, and we tested interactions between feedback and the ASCs to assess whether feedback attenuated label‐driven choices. We also assessed whether preferences differed by recruitment source (DUHS vs. national panel) by estimating a pooled conditional logit with site‐by‐attribute and site‐by‐opt‐out interactions, plus a three‐way interaction for the feedback effect on opting out (DUHS x Feedback x Optout). We then estimated site‐stratified models. We report (i) a joint Wald test of all site interactions to assess whether to pool the two recruitment sources across attributes and opt‐out, and (ii) a Wald test of DUHS x Feedback x Optout to assess whether the feedback effect differs by site. Full estimation details and results are provided in the Supporting Information S1.

Second, we investigated the impact of providing feedback on choice consistency using two different models. We allowed the error variance to systematically differ between the FEEDBACK and NOFEEDBACK groups. Changes in error variance represent differences in choice consistency^2^ (Börger 2016; DeShazo and Fermo 2002). We then estimated a heteroscedastic multinomial logit (HMNL) model on the data pooled across the FEEDBACK and NOFEEDBACK groups (Equation 4), with error variance specified to be a function of experimental manipulation (a binary variable ($F_{n}$), indicating the feedback mechanism) (Hole 2006; Swait and Adamowicz 2001b).

(4) $$V^{POOLED}=\lambda_{n}\left(\beta_{1}{\mathrm{ASC}}_{\mathrm{NO}\;\mathrm{DEVICE}}+\beta_{2}\text{PHYSICAL}\;\text{FUNCTIONING}+\beta_{3}R\text{ISK}\;\text{OF}\;\text{DEATH}+\beta_{4}\text{RISK}\;\text{OF}\;\text{COMPLICATIONS}+\beta_{5}\text{REMOTE}\;\text{PROGRAMMING}\right)$$

$$\lambda_{n}=\exp\left(\alpha F_{n}\right)$$

The scale parameter^3^ (in this case, associated with feedback) is indicative of the variance in unobserved utility. A negative sign indicates that feedback increases this variance, thereby reducing choice consistency.

The HMNL has well‐known limitations for our purposes: it cannot capture unobserved preference heterogeneity and, because taste and error‐scale are confounded, it can mask how feedback alters choice consistency (DeShazo and Fermo 2002; Swait and Louviere 1993). To address these issues and make the scale effect explicit, we estimate a heteroscedastic latent class (HLC; also termed a scale‐adjusted latent class) model in which feedback enters the scale‐class membership function as a determinant of choice consistency (Magidson and Vermunt, 2007; Fiebig et al. 2010).

Let $S_{nt}$ denote the choice set, that is, the set of alternatives available to respondent n in choice task t. In our case, $S_{nt}=\left\{\mathrm{Device}\;A,\mathrm{Device}\;B,\mathrm{No}\;\mathrm{Device}\right\}$.

Let $y_{ntj}=1$ if respondent n chose alternative j in task t, and $y_{ntj}=0$ otherwise. This binary indicator variable allows the choice probability expressions to be incorporated into the likelihood function in the usual way by raising the probability of each chosen alternative to the power of $y_{ntj}$.

We index alternatives by j and $j^{\prime }$, reserving k for attributes. The HLC (Magidson and Vermunt, 2007) assumes that the population can be decomposed in two overlapping ways: into S scale classes with class‐specific scale parameters ${\left\{\mu_{s}\right\}}_{s=1}^{S}$, and into M taste classes with class‐specific preference vectors ${\left\{\beta_{m}\right\}}_{m=1}^{M}$ (Magidson and Vermunt, 2007; Greene and Hensher 2003). This implies that, despite sharing the same coefficients within the same taste class, some respondents may display a different level of choice consistency, thereby belonging to different scale classes.

The systematic utility of alternative j for respondent n in task t, conditional on scale class s and taste class m, is $V_{ntj}|s,m=\mu_{s}\beta_{m}^{\prime }X_{ntj}$, so that the scale parameter multiplies the entire systematic utility of each alternative; where $\mu_{s}$ is the scale parameter for scale class s and $\beta_{m}$ is the taste parameter vector for taste class m.

The probability of choosing alternative $j\in S_{nt}$ in task t by respondent n, conditional on scale class s and taste class m, is given by:

(5) $$P\left(y_{ntj}|s,m\right)=\frac{\exp\left(\mu_{s}\beta_{m}^{\prime }X_{ntj}\right)}{\sum_{j^{\prime }\in S_{nt}}\exp\left(\mu_{s}\beta_{m}^{\prime }{X^{\prime }}_{ntj}\right)},j\in S_{nt}$$

Here, j and $j^{\prime }$ index alternatives within the choice set $S_{nt}$, while S denotes the number of scale classes in the latent class structure. The subscript in $\mu_{s}$ emphasizes that scale can vary across classes but is fixed within each class.

As in standard latent class models, class memberships are probabilistic. We allow distinct covariate sets for scale class and taste class membership, $z_{1n}$ and $z_{2n}$. The **scale‐class** membership probability is

(6) $$P\left(s|z_{1n}\right)=\frac{\exp\left(\gamma_{s}^{\prime }z_{1n}\right)}{\sum_{s^{\prime }=1}^{S}\exp\left(\gamma_{s^{\prime }}^{\prime },z_{1n}\right)},\;s=1,\text{…},S,$$

and the taste‐class membership probability is

(7) $$P\left(m|z_{2n}\right)=\frac{\exp\left(\theta_{m}^{\prime }z_{2n}\right)}{\sum_{m^{\prime }=1}^{M}\exp\left(\theta_{m}^{\prime },z_{2n}\right)},\;m=1,\text{…},M.$$

Here, $\gamma_{s}$ and $\theta_{m}$ are vectors of coefficients associated with $z_{1n}$ and $z_{2n}$, respectively. In our application, $z_{1n}$ includes a feedback indicator to capture how receiving feedback affects choice consistency (scale), while $z_{2n}$ includes demographics or survey characteristics influencing tastes (Fiebig et al. 2010; Greene and Hensher 2003).

The binary indicator $y_{ntj}$ is used in the likelihood function by raising the probability of each alternative to the power of $y_{ntj}$. The likelihood of the observed choices for respondent n is:

(8) $$P\left(y_{n}|z_{n}\right)=\sum_{s=1}^{S}P\left(s|z_{1n}\right)\sum_{m=1}^{M}P\left(m|z_{2n}\right)\prod_{t=1}^{T}\prod_{j=1}^{J}{\left[P\left(y_{ntj}|s,m\right)\right]}^{y_{ntj}}.$$

The overall log‐likelihood function for all respondents is then:

(9) $$lnL=\sum_{n=1}^{N}\ln\left[P\left(y_{n}|z_{n}\right)\right]=\sum_{n=1}^{N}\ln\left\{\sum_{s=1}^{S}P\left(s|z_{1n}\right)\sum_{m=1}^{M}P\left(m|z_{2n}\right)\prod_{t=1}^{T}\prod_{j=1}^{J}{\left[P\left(y_{ntj}|s,m\right)\right]}^{y_{ntj}}\right\}.$$

### 3.1 Estimation and Inference

Parameters ${\left\{\mu_{s}\right\}}_{s=1}^{S}$, ${\left\{\beta_{m}\right\}}_{m=1}^{M}$, and membership coefficients ${\left\{\gamma_{s}\right\}}_{s=1}^{S}$, ${\left\{\theta_{m}\right\}}_{m=1}^{M}$ are estimated by maximum likelihood. The HLC specification separates taste and scale heterogeneity, mitigating the scale‐taste confound (Swait and Louviere 1993), and provides a direct test of whether feedback shifts choice consistency (through $z_{1n}\to \gamma_{s}$) vs. shifting tastes (via $z_{2n}\to \theta_{m}$).

We also evaluated observed preference heterogeneity by incorporating respondent characteristics into the models. Covariates included age, sex, marital status, education, health literacy (measured by confidence in filling out medical forms),^4^ clinical history (e.g., prior heart attack, surgery, implanted device), recruitment source (panel vs. DUHS), response time (indicators for fast and slow completion), comprehension performance (indicators for achieving at least 8 and at least 9 comprehension questions correctly), and stated task experience (being very certain about choices; finding it very easy to answer the choice questions). These were chosen based on their potential to systematically influence the propensity to select the no‐device option. To capture systematic variation in choice consistency, we also modeled scale (error variance) as a function of feedback exposure and respondent characteristics. In the latent class models, sociodemographic and clinical covariates were introduced into the class membership function, while feedback was retained as a covariate in the scale component alongside response‐time, comprehension, literacy, certainty, and difficulty indicators.

## 4 Results

**FIGURE 3:**
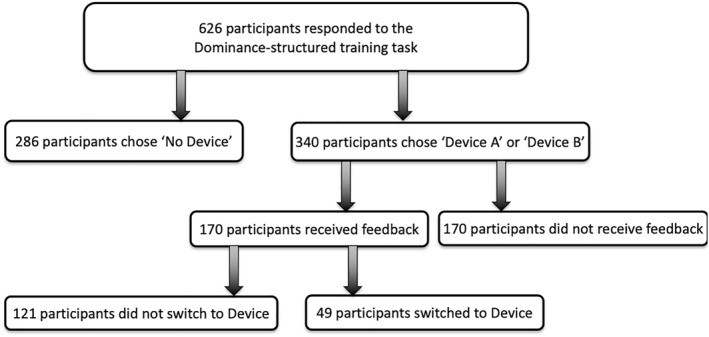
Flowchart of respondents through the experiment.

Figure 3 presents the flowchart of respondents through the experiment, detailing sample allocation across study arms. We found no statistically significant difference between respondents in the FEEDBACK and NOFEEDBACK groups in terms of socio‐demographic characteristics. The analysis also revealed no statistically significant differences in self‐reported health literacy measures, task difficulty, and response certainty between respondents who received feedback and those who did not (Table 2). Hence, any difference in results across the two subsamples is likely to be attributable to the experimental manipulation (i.e., the provision of feedback), not to differences in the underlying characteristics of the survey participants.

**TABLE 2:** Respondents' characteristics and responses among those who initially failed the dominated‐choice task.

	FEEDBACK (*N* = 170)	NOFEEDBACK (*N* = 170)	*p*‐value
Age			0.369^1^
Mean (SD)	63.23 (11.58)	64.37 (11.82)	
Response Time			0.511^1^
Mean (SD)	28.29 (26.01)	26.68 (18.54)	
Gender			0.329^2^
Male	89	80	
Female	81	90	
Education (what is the highest level of education you have completed?)			0.985^2^
Less than high school	3	1	
Some high school	4	3	
High school or equivalent	34	33	
Some college but not degree	42	40	
Technical school	13	12	
Associate's degree or 2‐year college degree	16	20	
4‐year college degree	32	32	
Some graduate school	6	7	
Graduate or professional degree	20	22	
Marital status (*what is your marital status?)*			0.234^2^
Single/never married	25	20	
Married/living as married	102	93	
Divorced/separated	31	34	
Widowed/surviving partner	12	21	
Other	0	2	
Ethnicity			0.187^2^
Hispanic, latino	10	5	
Not hispanic, latino	160	165	
Health literacy			
*How often do you have problems learning about your medical conditions because of difficulty understanding written information?*			0.542
Never	67	82	
Occasionally	57	51	
Sometimes	31	28	
Often	9	6	
Always	5	3	
*How confident are you filling out medical forms by yourself?*			0.127
Extremely	91	98	
Quite a bit	39	49	
Somewhat	25	13	
A little bit	12	6	
Not at all	3	3	
*How often do you have someone help you read hospital materials?*			0.129
Never	95	109	
Occasionally	40	36	
Sometimes	16	13	
Often	12	3	
Always	7	9	
Perceived task difficulty (*how did you find making choices between device options?*)			0.424^2^
Very easy	25	20	
Somewhat easy	46	57	
Neither easy nor difficult	33	39	
Somewhat difficult	61	48	
Very difficult	5	6	
Task certainity (*how certain you are about the choices you made?*)			0.328^2^
Very certain	44	57	
Somewhat certain	90	79	
Neither certain nor uncertain	18	16	
Somewhat uncertain	16	18	
Very uncertain	2	0	

*Note:* 1. *T*‐test of mean equality; 2. Pearson's Chi‐squared test with Yates' continuity correction.

In the dominance‐structured training task, 54% of respondents selected a device with associated risks and no benefits. Among those randomized to receive feedback (*n* = 170), 71% (*n* = 121) continued to choose a device after the feedback explicitly stated that the device had no benefit and added risk. In other words, despite being explicitly informed that the device provided no benefit and only added risk, a large proportion persisted with the device choice. ASCL estimates on the full sample confirmed positive and significant ASCs for “Device A” and “Device B” relative to “No Device” (Supporting Information S1: Table S2), indicating label utility. In the feedback subsample, feedback significantly reduced the ASCs for both “Device A” and “Device B” ($\text{joint}\;\text{test}\;\chi^{2}\left(2\right)=9.46,p=0.01$), consistent with feedback attenuating label‐driven preferences (Supporting Information S1: Table S2).

The results from the pooled data of the FEEDBACK and NOFEEDBACK groups (*n* = 340) show that all utility coefficients have the expected signs, confirming the theoretical validity of the model (Supporting Information S1: Table S1). Supporting Information S1: Table S1 presents the ASCL estimates for the full sample (Model 1) and the feedback subsample with feedback interactions (Model 2), including clustered standard errors, model diagnostics, and joint tests. Using an alternative‐specific conditional logit model, we found strong and internally consistent attribute effects: 1‐year NYHA improvements increased utility, while higher 30‐day mortality and complication risks decreased utility, remote adjustment increased utility (Table 3). In the full sample, both “Device A” and “Device B” exhibited positive alternative‐specific constants relative to “No Device” (${\mathrm{ASC}}_{\mathrm{Device}\;A}=0.449$,  SE = 0.106,  *p* < 0.01; ${\mathrm{ASC}}_{\mathrm{Device}\;B}=0.537$, SE = 0.112, *p* < 0.01), supporting label utility beyond attributes. In the feedback subsample, interacting ASCs with the feedback indicator yielded significant negative shifts for both devices (i.e., feedback significantly reduced both label utilities ($\mathrm{Feedback}\times {\mathrm{ASC}}_{\mathrm{Device}\;A}=-0.729$, SE = 0.249, *p* < 0.01; $\mathrm{Feedback}\times {\mathrm{ASC}}_{\mathrm{Device}\;B}=-0.748$, SE = 0.245, *p* < 0.01). A joint Wald test rejects the null of no feedback shift $\left(\chi^{2}\left(2\right)=9.46,p=0.009\right)$. The mean predicted probability of choosing “No device” increased from 0.096 without feedback to 0.180 with feedback (Supporting Information S1: Figure S1), an absolute rise of 0.0846 (8.46% points; cluster‐bootstrap 95% CI: 0.0832–0.0859; *n* = 340). This pattern was consistent with the negative feedback interactions on the device ASCs in Supporting Information S1: Table S1, indicating that feedback attenuated residual label utility and shifted choices toward the opt‐out (No Device). Together, these findings confirm the presence of label effects and demonstrate that the feedback prompt attenuated, but did not eliminate, label‐driven utility.

**TABLE 3:** Multinomial logit (preference‐space) with feedback in scale: utility and scale parameters.

Panel A. Utility coefficients (choice model)	Coef.	SE	z‐score	*p*‐value
Opt‐out (no device) versus device	−0.828	0.307	−2.70	0.007
Device ASC (alternative‐specific constant)	0.176	0.061	2.91	0.004
Physical functioning				
1‐year gain to NYHA class II (vs. none)	0.720	0.087	8.25	< 0.001
1‐year gain to NYHA class III (vs. none)	0.540	0.081	6.70	< 0.001
30‐day mortality risk (vs. 0%)				
2%	−0.228	0.082	−2.77	0.006
5%	−0.605	0.088	−6.87	< 0.001
10%	−1.204	0.123	−9.81	< 0.001
15%	−1.351	0.129	−10.48	< 0.001
In‐hospital complication risk (vs. 0%)				
5%	−0.195	0.082	−2.38	0.017
15%	−0.617	0.089	−6.97	< 0.01
40%	−1.067	0.100	−10.73	< 0.01
Remote device adjustment: Yes (vs. no)	0.178	0.052	3.41	< 0.01
Opt‐out x feedback	0.487	0.178	2.73	0.01
Opt‐out x respondent characteristics				
Female	0.195	0.153	1.28	0.201
Older	0.020	0.180	0.11	0.913
Married	−0.084	0.147	−0.57	0.567
High‐school or less	0.302	0.187	1.62	0.106
No college	0.153	0.260	0.59	0.557
No degree	−0.334	0.246	−1.35	0.176
Graduate degree	−0.191	0.261	−0.73	0.463
Known device type	0.210	0.150	1.40	0.160
Prior heart attack	−0.245	0.170	−1.44	0.151
White	−0.586	0.162	−3.61	< 0.01
Retired	0.441	0.180	2.45	0.014
Prior surgery	0.203	0.190	1.07	0.287
Disabled	0.020	0.174	0.12	0.907
NYHA I/II symptoms	−0.801	0.172	−4.65	< 0.01
Arrhythmia	−0.346	0.223	−1.55	0.120
Previous device implanted	−0.628	0.196	−3.21	< 0.01

Panel B. Scale (error variance) covariates	Coef.	SE	z‐score	*p*‐value
Feedback in scale	−0.272	0.104	−2.62	0.01
Model diagnostics				
LL (convergence)	−2381.0995			
McFadden's pseudo‐R^2^	0.112			
AIC/*n*	1.773			
BIC/*n*	1.838			
Number of choices	2720			
Number of respondents	340			
Number of parameters	30			

*Note:* Table shows preference parameters (Panel A) and covariates of scale (Panel B). Positive coefficients indicate higher utility relative to the base alternative; negative coefficients indicate lower utility. Feedback significantly reduced scale (−0.272, *p* = 0.01), meaning that choices became less consistent after the dominated‐task prompt.

In the pooled model with site interactions (Supporting Information S1: Table S2), attribute effects retain the expected signs. Only one site interaction (NYHA II) reached significance; the joint test of all site interactions was not significant $\left(\chi^{2}\left(11\right)=8.10,p=0.705\right)$, indicating no systematic site‐based taste heterogeneity. The feedback‐induced increase in the utility of opting out was positive and significant (Feedback×Optout=0.799;SE=0.267;p<0.01) and did not differ by site ($\mathrm{DUHS}\times \mathrm{Feedback}\times \mathrm{Optout}:\chi^{2}\left(1\right)=0.33;\;p=0.568$). Stratified models show a positive but imprecise estimate in the smaller DUHS sample (Panel S3‐A) (0.446; SE = 0.561; *p* = 0.427) and a significant effect in the panel sample (Panel S3‐B) (0.799; SE = 0.267; *p* < 0.01); the pooled interaction test confirms that the cross‐site difference is statistically negligible. Model‐based contrasts summarizing these site effects are provided in Supporting Information S1: Table S4. These findings support pooling across sites while reporting site robustness checks.

Table 3 reports the results from an HMNL model that examines how feedback affects the consistency of choices, including the feedback interaction on the No‐Device option for completeness. The HMNL model with covariates revealed that feedback significantly reduced choice consistency. The coefficient for Feedback x No‐Device was positive and significant (0.487, SE = 0.178, *p* = 0.01), corroborating the finding that feedback increased the likelihood of choosing the opt‐out.^5^ Several covariates were also strongly associated with the probability of opting out (Table 3). Respondents with a baseline NYHA class I‐II diagnosis (coef. = −0.801, *p* < 0.01) or a previous implanted device (coef. = −0.628, *p* < 0.01) were markedly less likely to choose the no‐device option. Retired respondents (coef. = 0.441, *p* = 0.014) were more likely to opt out, whereas respondents identifying as White (coef. = −0.586, *p* < 0.01) were less likely to do so. Other covariates, such as sex, age, and education level, were not statistically significant. Importantly, the scale covariate for feedback remained negative (−0.272, SE = 0.104, *p* = 0.01), indicating that even after accounting for the preference shift, feedback recipients exhibited approximately 24% lower choice consistency. The HMNL model confirms that the feedback intervention had two effects: it increased respondents' tendency to opt‐out, and it increased randomness in their choices.

We also estimated an extended HMNL model incorporating additional covariates of scale to account for further sources of heterogeneity (e.g., response time metrics, certainty ratings). The results (reported in Supporting Information S1: Table S5) showed that the Feedback x No‐Device interaction remained positive and significant (0.344, SE = 0.154, *p* = 0.025) and that the scale effect for feedback remained negative and significant (−0.284, SE = 0.108, *p* = 0.01), confirming approximately 25% lower consistency. Other scale covariates, such as comprehension performance (indicators for achieving at least 8 comprehension questions correctly) (0.291, SE = 0.096, *p* < 0.01) and health literacy (measured by confidence in filling out medical forms) (−0.631, SE = 0.211, *p* < 0.01), were also significant, highlighting additional factors influencing choice consistency.

Table 5 presents the HLC model results, where we identified three latent classes based on Bayesian information criterion (BIC) and Akaike information criterion (AIC) statistics, parsimony, and the plausibility of the results. Posterior class shares were 63.3% for Class 1, 20.9% for Class 2%, and 15.8% for Class 3. Class 1 (“Pro‐device”) represented the majority of respondents. This group valued improvements in physical functioning (NYHA II: 0.707, *p* < 0.01; NYHA III: 0.572, *p* < 0.01) and was averse to higher 30‐day mortality and complication risks, with the expected monotonic ordering (e.g., mortality 5%: −0.233, 10%: −0.614, 15%: −0.775; *p* < 0.01). The opt‐out constant was strongly negative (−3.728, *p* < 0.01), indicating a clear preference for receiving a device over “no device.” In the scale equation, the feedback prompt had no detectable effect (−0.064, *p* = 0.56), suggesting no influence on choice consistency. Several process variables, such as comprehension performance (indicators for achieving at least 8 comprehension questions correctly) (1.028, *p* < 0.01) and stated task certainty (very certain about choices) (“very certain” = 0.417, *p* < 0.01) were linked to more consistent choices. In the class‐membership model, White race and NYHA I/II symptoms were positively associated with belonging to Class 1 (“Pro‐device”).

**TABLE 4:** Heteroskedastic latent class logit, pooled FEEDBACK and NO‐FEEDBACK (*n* = 340).

	Class 1 ‐ “pro‐device”	Class 2 ‐ “pro‐device, risk‐sensitive”	Class 3 ‐ “anti‐device”
Preference parameters (utility)			
1‐year gain, NYHA II	0.707 (0.101) ***	0.638 (0.160) ***	0.609 (0.275) **
1‐year gain, NYHA III	0.572 (0.091) ***	0.423 (0.119) ***	0.209 (0.227)
30‐day mortality (vs. 0%)			
2%	−0.038 (0.069)	−0.459 (0.165) ***	−0.844 (0.281) ***
5%	−0.233 (0.080) ***	−1.024 (0.257) ***	−1.219 (0.339) ***
10%	−0.614 (0.132) ***	−2.682 (0.689) ***	−2.140 (0.630) ***
15%	−0.775 (0.129) ***	−3.243 (0.868) ***	−1.746 (0.528) ***
In‐hospital complications (vs. 0%)			
5%	−0.169 (0.071) **	0.639 (0.167) ***	−0.947 (0.282) ***
15%	−0.440 (0.088) ***	−0.409 (0.172) **	−1.790 (0.437) ***
40%	−0.875 (0.120) ***	−0.734 (0.228) ***	−1.409 (0.412) ***
Remote adjustment (vs. no)	0.255 (0.050) ***	0.013 (0.054)	−0.081 (0.187)
Opt‐out (no device)	−3.728 (0.502) ***	−1.288 (0.344) ***	0.292 (0.332)
ASC: No device	0.129 (0.049) ***	−0.004 (0.089)	0.483 (0.224) **
Explanatory variables of scale			
Feedback (vs. no)	−0.064 (0.111)	−1.718 (0.518) ***	0.501 (0.209) **
At least 8 comprehension questions correct	1.028 (0.158) ***	1.023 (0.288) ***	−0.147 (0.269)
At least 9 comprehension questions correct	−0.818 (0.204) ***	2.572 (0.616) ***	−0.212 (0.485)
Response time (fast completion)	−0.179 (0.143)	−1.540 (0.510) ***	0.592 (0.334) *
Response time (slow completion)	−0.223 (0.135) *	0.300 (0.367)	0.268 (0.228)
DUHS site	0.104 (0.143)	2.275 (0.569) ***	−1.077 (0.307) ***
Confidence filling out medical forms	−0.457 (0.216) **	2.655 (0.686) ***	−0.372 (0.375)
Very certain about the choices made (vs. not certain)	0.417 (0.131) ***	−0.311 (0.300)	−0.232 (0.346)
Very easy to answer the choice questions (vs. no)	−0.317 (0.172) *	0.877 (0.555)	−0.378 (0.444)
Covariates of class membership			
Constant	0.855 (0.683)	−1.021 (0.934)	0 (fixed)
Feedback	−0.521 (0.351)	0.221 (0.472)	—
Female	−0.243 (0.375)	−0.479 (0.481)	—
Older	−0.209 (0.442)	0.101 (0.559)	—
Married	0.162 (0.362)	−0.054 (0.467)	—
HS Or less	−0.422 (0.473)	0.022 (0.587)	—
No college	−0.365 (0.577)	0.081 (0.910)	—
Non‐degree	0.663 (0.552)	1.056 (0.841)	—
Graduate	0.523 (0.728)	1.911 (0.906) **	—
Known type of heart failure (HEpEF or HFrEF)	−0.194 (0.377)	0.589 (0.492)	—
Prior heart attack	−0.201 (0.387)	−0.553 (0.514)	—
White	0.889 (0.409) **	0.470 (0.507)	—
Retired	−0.430 (0.442)	−0.130 (0.576)	—
Prior surgery	0.173 (0.464)	−0.786 (0.690)	—
Disability	0.047 (0.424)	0.155 (0.570)	—
NYHA I/II symptoms	0.851 (0.397) **	0.916 (0.508) *	—
Arrhythmia	0.016 (0.475)	−1.477 (0.754) *	—
Prior implanted device	0.476 (0.460)	1.072 (0.682)	—
Class share (%)	63.31 (2.90) ***	20.92 (2.68) ***	15.77 (2.12) ***
Model diagnostics			
LL (convergence)	−1959.9996		
McFadden's pseudo‐R^2^	0.2694		
AIC/*n*	1.5140		
BIC/*n*	1.7290		
Number of choices	2720		
Number of respondents	340		
Number of parameters	99		

*Note:* Standard errors in parentheses; *, **, *** denote 10%, 5%, and 1%. “Scale” rows report log‐scale coefficients; positive values imply larger scale (lower error variance) conditional on class. Class‐membership coefficients are relative to Class 3 (normalization). About 63% of respondents were predicted to be members of the first latent class, and 21% of respondents were predicted to be members of the second latent class. The third class represented a strongly anti‐device group, representing preferences of about 16% of the sample likely to be members of this group. Class 1 (pro‐device): Logically ordered preferences for benefits and risks. Prefers average device over no device. Class 2 (pro‐device, risk‐sensitive): cares more about risks. Prefers average device over no device. Class 3 (anti‐device): Strongly prefers no device over average device, despite benefit‐risk profiles offered.

Abbreviations: AIC = Akaike Information Criterion; BIC = Bayesian Information Criterion; *n* = Number of observations; NYHA = New York Heart Association.

**TABLE 5:** Within‐class wald tests for monotone risk ordering (one‐sided). H_0_: adjacent differences satisfy expected order.

Class	Attribute	Adjacent difference	z	p (one‐sided)	Adjacent difference	z	p (one‐sided)	Joint IUT p	Hypothesis supported?
1	Mortality risk (2→5→10→15)	Δ (2→5) = 0.195	2.59	< 0.01***	Δ (5→10) = 0.381	3.45	< 0.01***	0.11	Not supported (fails at 10→15)
	Complication risk (5→15→40)	Δ (5→15) = 0.272	3.46	< 0.01***	Δ (15→40) = 0.435	5.16	< 0.01***	< 0.01***	Supported
2	Mortality risk (2→5→10→15)	Δ (2→5) = 0.565	3.37	< 0.01***	Δ (5→10) = 1.657	3.37	< 0.01***	0.20	Not supported (fails at 10→15)
	Complication risk (5→15→40)	Δ (5→15) = 1.048	3.59	< 0.01***	Δ (15→40) = 0.325	2.10	0.02**	0.02**	Supported
3	Mortality risk (2→5→10→15)	Δ (2→5) = 0.376	1.32	0.09*	Δ (5→10) = 0.921	1.72	0.04**	0.74	Not supported (fails at 10→15)
	Complication risk (5→15→40)	Δ (5→15) = 0.843	2.33	0.01**	Δ (15→40) = −0.380	−1.04	0.85	0.85	Not supported (fails at 15→40)

*Note:* *, **, *** indicate statistical significance at the 10%, 5%, and 1% levels, respectively. The “Joint IUT *p*” column reports intersection‐union test results across adjacent risk levels within each class. The monotonicity hypothesis is considered supported only if all adjacent differences follow the expected order at conventional significance levels.

Class 2 (“Pro‐device, risk‐sensitive”) accounted for roughly one‐fifth of respondents. As in Class 1, this group valued gains in functioning (NYHA II: 0.638, *p* < 0.001; NYHA III: 0.423, *p* < 0.01), but showed strong aversion to mortality risk (−0.459, −1.024, −2.682, −3.243 for 2%, 5%, 10%, 15%, respectively; *p* < 0.01). The opt‐out constant was negative (−1.288, *p* < 0.01), indicating a preference for a device on average, albeit less strongly than Class 1. The key result for this class is the large, negative feedback coefficient in the log‐scale equation (−1.718, *p* < 0.01), indicating that the feedback prompt was associated with decreased scale and increased error variance (i.e., less consistent choices). Comprehension performance (indicators for achieving at least 8 and 9 comprehension questions correct) (e.g., at least 8 correct questions = 1.023, *p* < 0.01; at least 9 correct questions = 2.572, *p* < 0.01), survey completion speed (fast completion = −1.540, *p* < 0.01), the DUHS site (2.275, *p* < 0.01), and health literacy (measured by confidence in filling out medical forms) (2.655, *p* < 0.01) were linked to choice consistency. In the class‐membership equation, graduate education was positively associated with belonging to Class 2 (1.911, *p* = 0.035), while having arrhythmia was negatively associated (−1.477, *p* = 0.050).

Class 3 (“Anti‐device”) was the smallest segment (15.8%). This group had strong negative preferences for mortality risk and complications (e.g., mortality 5%: −1.219, *p* < 0.01; 10%: −2.140, *p* < 0.01; 15%: −1.746, *p* < 0.01; complications 15%: −1.790, *p* < 0.01; 40%: −1.409, *p* < 0.01). Unlike the other classes, this class was indifferent to No Device (0.292, *p* = 0.38). The most notable process finding is that feedback was associated with increased scale for this class (0.501, *p* = 0.016), implying more consistent choices after the feedback prompt.

**FIGURE 4:**
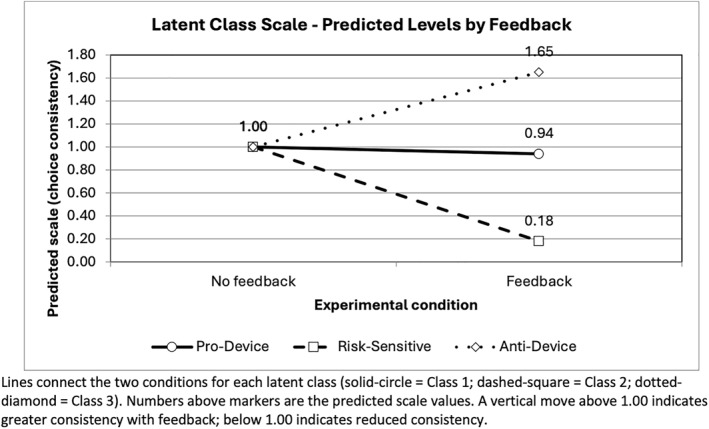
Class‐specific feedback effects on choice consistency (scale factors) in the latent class model.

Figure 4 illustrates the class‐specific effects of feedback on choice consistency, as captured by the latent class scale factors. In the majority “pro‐device” class (Class 1, 63.3%), feedback had a negligible impact on consistency, with the estimated scale factor close to unity (0.94). In contrast, respondents in Class 2 (20.9%) became markedly less consistent after receiving feedback, with their scale factor falling to 0.18. This suggests that the feedback prompt disrupted compensatory processing, yielding more random responses. By comparison, respondents in Class 3 (15.8%) exhibited a strong positive response to feedback, with their scale factor increasing to 1.65. For this smaller group, feedback appears to have clarified decision strategies and reinforced systematic aversion to the device. These results therefore highlight the heterogeneity masked by the average HMNL effect, which indicated a general reduction in choice consistency. The latent class results suggest that feedback disrupted choice patterns for some respondents while making others more consistent in their decision rules and choices.

Regarding risk ordering within classes, the majority class (Class 1) showed the expected monotonic pattern across risk levels^6^. The smaller classes displayed a few preference reversals (e.g., the positive coefficient for 5% complications in Class 2; the 15% vs. 10% mortality comparison in Class 3). Such reversals are not unexpected. Because class‐specific estimates are based on posterior membership weights, smaller class sizes carry larger standard errors, and occasional reversals are common in latent‐class applications even when the underlying preferences are monotonic (Hensher et al. 2012; Train 2009).

## 5 Discussions

The objective of the present study was to examine whether providing real‐time feedback during a training task in a DCE could influence choice behavior. We empirically investigated this by comparing a group of respondents who received feedback to those who did not and restricting the analysis to respondents who chose a device with risks but no benefits. We evaluated two hypotheses: first, that feedback would increase choice consistency by reducing error variance; and second, that it would reduce the likelihood of selecting a device, thereby nudging respondents toward the opt‐out (i.e., no device) option.

Our results provide mixed support for these hypotheses. With respect to Hypothesis 1 (Consistency), the results contradicted expectations. On average, feedback decreased choice consistency, with recipients' choices showing 25% higher error variance compared to those who received no feedback. This change is meaningful, indicating a sizable reduction in how consistently respondents traded off attributes after receiving feedback. However, the effect was not uniform, with the latent class model revealing substantial heterogeneity. Feedback had no measurable impact on consistency for the majority “Pro‐device” class, reduced consistency sharply for the “Pro‐device, risk‐sensitive” class, and increased consistency for the smaller “Anti‐device” class. These heterogeneous findings suggest that feedback altered cognitive processing in different ways across subgroups, clarifying decision strategies for some respondents while disrupting them for others.

For Hypothesis 2 (Behavioral adjustment), the results aligned more closely with predictions. Respondents who received feedback were significantly more likely to select the opt‐out (“No Device”) option in subsequent choice sets. In the choice questions, 73% of feedback recipients chose “No Device” at least once, compared to 58% in the no‐feedback group (difference of 15% points). These changes are large in the context of discrete choice behavior, suggesting the feedback intervention had a meaningful impact on decision patterns.

Our findings have a number of practical implications for DCE practitioners. First, they demonstrate that training tasks with feedback are not neutral interventions. Rather than simply improving comprehension, feedback can act as an intervention that changes both *how* people choose (consistency) and *what* they choose (preference patterns). This dual effect was captured by including feedback in both the utility and scale functions of our models. This dual influence underscores the need for careful consideration of how feedback is introduced and how its effects are modeled.

Second, an important implication of our findings is the need to reconsider what it means to present respondents with a dominance‐structured training task. Although our training task was structured so that the device option offered no benefit but carried risks, it is unlikely that respondents viewed it as truly dominated. In practice, no profile is ever completely stripped of unobserved or inferred characteristics. Respondents may attach value to the label (“Device A” or “Device B”), infer unmeasured benefits from the mere presence of a device, or rely on action‐inaction heuristics that favor intervention over doing nothing (Johnson et al. 2019). These mechanisms likely explain why many respondents persisted in choosing risky device options with no benefits even after feedback was provided.

This observation is consistent with prior studies showing that 10%–20% of respondents in DCEs often select options that appear dominated from the researcher's perspective (Johnson et al. 2019; Veldwijk et al. 2023). Traditionally, these choices have been treated as errors and used as grounds for excluding respondents ex post (Jonker et al. 2022; Tervonen et al. 2018). Our findings challenge this practice. Rather than discarding these choices, we used them as an opportunity to test the effect of real‐time feedback. The findings suggest that feedback did alter subsequent behavior, but not uniformly: for some respondents, it clarified the task and redirected attention toward the attribute profiles, while for others it introduced confusion and reduced consistency.

Third, the persistence of device selections even after feedback highlights the importance of label utility in health‐related DCEs. Alternatives that represent “doing something” may be valued above and beyond their attribute levels (Bridges et al. 2011; De Bekker‐Grob et al. 2012; Hensher et al. 2012). In our pooled models, device alternatives carried positive constants, consistent with respondents attributing inherent value to intervention. When feedback was introduced, these constants shifted downward and the predicted probability of opting out increased, indicating that the prompt encouraged respondents to rely more on attribute information. Yet, labels and heuristics continued to exert influence, suggesting that deeply held beliefs or preferences cannot be easily overridden by corrective feedback.

Conceptually, our findings speak to a debate about the rational‐choice view that dominated options should never be chosen and behavioral accounts that emphasize bounded rationality and framing. Tversky and Kahneman argue that dominance and invariance are central rationality requirements, yet also document systematic framing effects and other violations in real decisions (Tversky and Kahneman 1986). In health‐care DCEs, Ryan and Bate show that the assumptions of rationality, continuity, and symmetry are sometimes violated even when choices remain interpretable (Ryan and Bate 2001), while Scott finds that a substantial share of respondents exhibit dominant or lexicographic preferences that conflict with standard compensatory models but nevertheless convey strong priorities over attributes (Scott 2002). Together with broader work on bounded rationality and fast‐and‐frugal heuristics (Gigerenzer and Goldstein 1996; Simon 1955), this literature suggests that what we label as “dominance failures” can arise because respondents simplify complex tasks using heuristics, adopt lexicographic preferences, or interpret labels and attributes through their own frames, rather than because they lack well‐defined preferences. Our study should therefore not be read as offering a definitive or “quintessential” account of dominated tasks or feedback in DCEs. Our perspective is pragmatic rather than doctrinal: we treat a dominance‐structured task as a useful benchmark for designing training, probing comprehension, and studying how feedback alters behavior within a clearly defined attribute frame, rather than as a universal standard for judging respondent rationality. The results are specific to one health condition, set of attributes, and one feedback format, and are intended to complement, not replace, other approaches that model heuristics, attribute non‐attendance, and alternative decision rules in health preference research (Erdem et al. 2014; A. Pearce et al. 2021; Ryan and Bate 2001; Scott 2002).

Fourth, a key question arising from our findings is why feedback reduced consistency. One plausible explanation is cognitive overload: the feedback, while intended as clarification, might have caused respondents to overthink or second‐guess their choices, introducing noise. Feedback Intervention Theory posits that feedback can sometimes shift attention away from the task toward the act of being evaluated (Kluger and DeNisi 1996), potentially weakening performance. In our case, some participants may have become preoccupied with not repeating a “mistake”, leading to more random responses subsequently. The latent class results illustrate this dynamic. For the “Pro‐Device, Risk‐Sensitive” group (Class 2), feedback seemed to act as a warning about risks, but their subsequent choices became less consistent, suggesting that they employed noncompensatory rules such as “avoid high risks at all costs”. On the other hand, respondents in the Anti‐Device class (Class 3) became more consistent after feedback, systematically rejecting device options. For these respondents, the feedback likely affirmed a latent skepticism they already had about the devices. These divergent patterns highlight that feedback can either disrupt or reinforce decision strategies depending on respondents' prior orientations. This heterogeneity aligns with findings in behavioral research showing that interventions rarely produce homogeneous outcomes; context and individual differences matter (Bandiera et al. 2015). In classic learning tasks, feedback typically improves performance on average, yet some learners benefit more than others. In our preference elicitation context, we essentially asked respondents to learn about their own preferences under guidance, a less straightforward task.

Our study is not exempt from limitations. First, the sample is restricted to heart failure patients from a national web panel and a single medical center, which may limit the generalizability of the findings to other populations. Second, while our experiment identified how respondents reacted to feedback, it did not capture why they made those choices. We did not collect qualitative data that could have provided insight into whether participants misunderstood the scenario, doubted the information, or simply valued the idea of a device despite its lack of benefits. We did not also systematically record verbatim reasons during pretesting, but the observed patterns supported the decision to include the dominance‐structured training task and feedback components to reduce unintended inconsistencies rather than to infer a genuine preference for a device when faced with a seemingly inferior option. As a result, we can only infer motivations, such as label effects, hope for unspecified benefits, misunderstanding of probabilities, or inertia to act, from patterns in the data rather than from respondents' own explanations. Future work should include qualitative elicitation alongside DCE tasks to identify mechanisms more clearly and to strengthen the interpretation of feedback effects. Third, our measures of health literacy and engagement relied on single‐item self‐reports and were incorporated in a simplified manner. While our research suggests these factors may play a role in feedback effects, more rigorous approaches, such as hybrid choice models or latent variable approaches, would allow richer integration of these constructs (Raaijmakers et al. 2019). Fourth, our DCE did not randomize the positional order of alternatives in the choice tasks. The opt‐out (No Device) option was always presented in the same position in the choice tasks, creating the potential for order effects. If respondents systematically favored or ignored options based on their placement, this could bias estimated utility for the No‐Device option. Because the ordering was fixed for all respondents, our comparisons between feedback versus no‐feedback groups remain valid. A recent systematic review concluded that most of the included studies documented statistically significant ordering effects and recommended mitigation through randomization of presentation orders and related design features (Boxebeld 2024). Future DCE studies on this topic should consider randomizing alternative positions to disentangle label effects from position effects. Fifth, although our results show that feedback can meaningfully alter choice patterns, the study and its implications are restricted to a specific feedback intervention in a single health condition (heart failure) and a specific set of attributes, and the results may differ for other conditions or attributes. They do not suggest that stated‐preference evidence is inherently unreliable. Our contribution is to show, in one intensively studied case, that feedback built around a dominance‐structured task can have sizable and heterogeneous effects on choice behavior. For decision makers, the key implication is that guidance and feedback elements within DCEs must be carefully justified, transparently reported, and, where feasible, empirically evaluated if stated‐preference evidence is to be combined with clinical and economic data in benefit‐risk assessment or reimbursement decisions (Benz et al. 2020; FDA 2016). Sixth, the study investigated only one type of feedback intervention. Future research should investigate how variations in the type, timing, format, or content of feedback could influence results. Further research should also explore alternative feedback mechanisms across different contexts and populations to develop best practices for incorporating feedback in DCEs. This could include experimentally varying the framing of feedback, for example, comparing neutral explanations with more directive prompts, to assess whether gentler forms of feedback improve choice consistency without distorting underlying preferences. Complementary qualitative follow‐up would also be important for elucidating respondents' thought processes. Some respondents may interpret feedback as helpful clarification, while others may experience it as corrective or even a punitive signal, leading to changes in decision strategy or disengagement. Understanding these heterogeneous reactions would clarify the conditions under which feedback serves as constructive guidance versus when it risks becoming a source of noise or misdirection into stated‐preference data. Finally, it is important to be transparent about how this experiment has been reported across publications. The original paper, published in a medical specialty journal with a clinician audience in mind (Reed et al. 2022), did not explicitly describe the existence of the feedback and no‐feedback arms. Current reporting guidance for DCEs emphasizes full disclosure of design features such as randomization and differential information provision for readers to assess the study and for other researchers to replicate or build on it (Bridges et al. 2011; Hauber et al. 2016; Ride et al. 2024). In our case, the feedback mechanism was originally conceived as a way to reduce unintentional selection of the dominated option, but omitting explicit mention of the two arms in the earlier substantive paper is not aligned with these evolving standards. By documenting the feedback design and examining its implications here, we aim both to share the findings and to offer a concrete reminder of why detailed reporting of DCE designs matters for scientific interpretation and advancement.

Beyond these design‐specific considerations, our study also speaks to a broader methodological debate about secondary analyses and multiple publications from the same dataset. More broadly, this debate is particularly relevant for DCEs in health economics. DCE datasets are increasingly being used to answer multiple, distinct research questions, particularly in health economics where DCEs are costly to field and often rich in behavioral content. As their use has expanded, concerns have shifted from “whether” to use DCEs toward “how” they are designed, analyzed, and reported. Recent reviews and bibliometric studies document substantial variation in DCE quality and highlight inadequate reporting of methodological details, such as attribute development, experimental design, and task structure, as a barrier to critical appraisal and policy use (Soekhai et al., 2019; Wang et al. 2021). Large cohort studies and clinical trials routinely generate several publications from a single protocol, and current guidance does not prohibit this practice provided that each paper addresses a clearly different question, is transparent about the shared data source, and avoids redundant overlap in analyses and conclusions (Altay and Koçak 2021). Major research funders and methodological guideline documents also argue that reusing existing data can increase the scientific and societal value of research by enabling validation of findings, addressing new questions, and maximizing the return on costly data‐collection efforts. The U.S. National Institutes of Health Data Management and Sharing Policy, for example, states that sharing scientific data “accelerates biomedical research discovery” by enabling validation of results, providing access to high‐value datasets, and “promoting the reuse of data for future research studies” (https://grants.nih.gov/policy‐and‐compliance/policy‐topics/sharing‐policies/dms/policy‐overview) (Smith et al. 2011). Editorial, guideline documents, and the metascience literature draw a clear distinction between duplicate or “salami‐sliced” publications, which fragment essentially similar results into several papers without adequate cross‐referencing, and legitimate secondary analyses that address substantively different questions or apply new methods (Altay and Koçak 2021; van Raaij 2018; Watson et al. 2015). In this literature, acceptable multiple publications from the same dataset are characterized by distinct research questions, different analytic focuses, and explicit acknowledgment of the relationship between articles, coupled with careful explanation of what is actually new (Altay and Koçak 2021; Fine and Kurdek 1994). In our case, the parent paper (Reed et al. 2022) reports the substantive benefit‐risk preferences for heart‐failure devices, whereas the present study focuses on a methodological sub‐experiment that randomised a real‐time feedback prompt in a single dominance‐structured task and examines how dominance‐structured feedback intervention shapes respondents' choice behavior. The behavioral mechanisms, model specifications, and outcome measures we examine are not revisitations of the same questions but extensions that could not be fully developed within the scope of the original article. In this sense, our analysis illustrates constructive data reuse that complements, rather than undermines, the validity of the original substantive paper, while recognizing that replication of feedback effects in independent DCEs would further strengthen the evidence base. Consistent with broader concerns about avoidable research waste, we view this type of carefully disclosed, non‐redundant secondary analysis as a way to increase the value extracted from costly preference studies, rather than to erode the validity of prior work (Chalmers and Glasziou 2009; Glasziou et al. 2014; Macleod et al. 2014; Salman et al. 2014).

## 6 Concluding Remarks

In conclusion, our study demonstrates that incorporating feedback into a DCE can significantly change respondent behavior, though not always in the intended way. Our findings highlight that respondents did not simply absorb feedback as neutral guidance; instead, they interpreted it in various ways, leading to heterogeneous effects. Some respondents became less consistent, perhaps due to confusion or overthinking prompted by the feedback, while others treated it as a cue to adjust behavior. Rather than universally improving choice quality, feedback in our experiment led to more random choices and a shift in preferences toward the opt‐out option. This suggests caution in interpreting interventions aimed at “guiding” respondents, as they might unintentionally introduce bias or noise. For DCE practitioners, adequate training before the DCE begins might be preferable to mid‐stream feedback that could be misconstrued as a “correct‐answer” cue. If feedback is to be used, it should be carefully designed and tested to ensure it clarifies rather than directs respondent choices. Finally, further research is needed to determine the generalizability of these results and to test different types of feedback (e.g., purely informational vs. advisory) across varied contexts (other health decisions or unlabeled choice designs) and populations. A better understanding of these dynamics will be key to developing best practices for improving respondent engagement and data quality without unintentionally biasing choices.

## Funding

Funding for the original study was coordinated by the Medical Device Innovation Consortium (MDIC) with contributions from Abbott, Abiomed, Boston Scientific, CVRx Inc., Edwards Life Sciences, Medtronic, and the US Food and Drug Administration via subcontract between Duke and MDIC (BAA‐00123) as part of MDIC's Framework for Patient Input in Medical Device Clinical Trials. This paper was partly funded by the Measurement and Regulatory Science (MaRS) Postdoctoral Fellowship Program at Duke University.

## Conflicts of Interest

The authors declare no conflicts of interest.

## Disclaimer

The U.S. Food and Drug Administration (FDA) participated as a member of MDIC SPI Heart Failure Working Group, but did not contribute to the drafting of this manuscript. The contents represent the views of the authors and do not necessarily represent the official views of, and are not an endorsement by, the U.S. FDA/Department of Health and Human Services (HHS) or the U.S. Government. The views, findings, and interpretations contained in this document do not constitute FDA guidance, position on this matter, or legally enforceable requirements. The MDIC SPI Heart Failure Working Group was deactivated after completing the work.

## Supporting information


Supporting information S1

## Data Availability

The authors have nothing to report.

## Endnotes

^1^
Note that participants who correctly chose the dominant option (“no‐device” option) did not receive feedback.

^2^
Choice consistency as measured by the scale parameter in Random Utility Models (RUM) is widely considered as an indicator for accuracy and choice quality (Evgeniou et al. 2005; Toubia et al. 2004). We interpret the scale parameter $\lambda_{n}=\exp\left(\alpha F_{n}\right)$ as a measure of choice consistency or certainty (Swait and Adamowicz 2001a). A higher scale (λ) implies lower error variance in the utility equation, meaning the respondent's choices are more consistent (closely aligned with the deterministic part of utility). Conversely, a lower scale indicates higher randomness (less consistency) in choices. Thus, if providing feedback affects λ, it is affecting how deterministically versus randomly respondents make choices.

^3^
The key parameters of interest for our hypotheses were (i) the feedback dummy in the scale function (α in $\lambda_{n}=\exp^{\left(\alpha F_{n}\right)}$), which tests whether feedback changes the scale/variance of choices, and (ii) the feedback interaction on the No‐Device utility ($\alpha_{feedback}$ in Equation 3), which tests whether feedback shifts preference for the opt‐out option. A priori, we expected α to be positive (feedback reducing error variance, thus α>0 would mean higher scale for feedback group, an improvement in consistency) and $\alpha_{feedback}$ to be possibly positive (feedback making opt‐out more attractive, if feedback acts as a nudge away from dominated devices). We used two‐sided significance tests for these effects at conventional levels (α=0.05).

^4^
The self‐reported health literacy indicators (originally 5‐point Likert scales) were dichotomized for analysis.

^5^
Providing feedback had a substantial effect on choices: for instance, based on our MNL estimates, the probability of choosing the No Device option in a typical choice scenario roughly doubled, from about 10% without feedback to about 20% with feedback, all else equal (this is implied by the feedback interaction coefficient of 0.765). Likewise, the 25% reduction in scale due to feedback corresponds to a large increase in randomness; in practical terms, feedback recipients made choices that were about half as predictable from the observed utility differences as those of non‐recipients.
