# Supplementary material for: Guidance or Misdirection? Unpacking the Role of Feedback in Health Preference Assessments
Source: Health Econ. 2026 Mar 3;35(6):910–28. doi: 10.1002/hec.70093 (PMC13126101; doi:10.1002/hec.70093)
Supplement: Supplementary file 1 — Supporting information S1 [file HEC-35-910-s001.docx]

**Supplementary Material**

**Table S1. Alternative-specific conditional logit (ASCL) estimates for pooled data and feedback subsample; No Device is the reference alternative.**

| **Attributes and levels** | **Coefficient** | **Standard error** | **Z-score** | **p-value** | **Coefficient** | **Standard error** | **Z-score** | **p-value** |
| --- | --- | --- | --- | --- | --- | --- | --- | --- |
|  |  |  |  |  |  |  |  |  |
|  |  |  |  |  |  |  |  |  |
|  | **Model 1: Full sample (N = 626)** |  |  |  | **Model 2: Feedback subsample with feedback terms (N = 340)** |  |  |  |
| **Physical functioning** |  |  |  |  |  |  |  |  |
| 1-year gain in NYHA class II | 0.797 | 0.060 | 13.351 | 0.000 | 0.634 | 0.079 | 8.030 | 0.000 |
| 1-year gain in NYHA class III | 0.601 | 0.054 | 11.155 | 0.000 | 0.462 | 0.073 | 6.307 | 0.000 |
| **30-day mortality risk (vs 0%)** |  |  |  |  |  |  |  |  |
| 2% | -0.204 | 0.047 | -4.383 | 0.000 | -0.211 | 0.061 | -3.460 | 0.001 |
| 5% | -0.554 | 0.058 | -9.559 | 0.000 | -0.534 | 0.079 | -6.745 | 0.000 |
| 10% | -1.173 | 0.101 | -11.651 | 0.000 | -1.074 | 0.132 | -8.117 | 0.000 |
| 15% | -1.338 | 0.094 | -14.209 | 0.000 | -1.200 | 0.118 | -10.157 | 0.000 |
| **In-hospital complication risk (vs 0%)** |  |  |  |  |  |  |  |  |
| 5% | -0.221 | 0.050 | -4.419 | 0.000 | -0.187 | 0.068 | -2.758 | 0.006 |
| 15% | -0.569 | 0.057 | -9.939 | 0.000 | -0.547 | 0.080 | -6.851 | 0.000 |
| 40% | -1.046 | 0.071 | -14.690 | 0.000 | -0.947 | 0.094 | -10.090 | 0.000 |
| Remote device adjustment (vs No) | 0.097 | 0.040 | 2.408 | 0.016 | 0.155 | 0.055 | 2.844 | 0.004 |
| **Alternative-specific constants (Device A is base)** |  |  |  |  |  |  |  |  |
| ASC: Device A (vs No device) | 0.449 | 0.106 | 4.235 | 0.000 | 1.469 | 0.205 | 7.171 | 0.000 |
| Feedback x ASC: Device A | - | - | - | - | -0.729 | 0.249 | -2.931 | 0.003 |
| ASC: Device B) (vs No device) | 0.537 | 0.112 | 4.788 | 0.000 | 1.637 | 0.210 | 7.783 | 0.000 |
| Feedback x ASC: Device B | - | - | - | - | -0.748 | 0.245 | -3.051 | 0.002 |

*Notes: NYHA = New York Heart Association. Robust standard errors clustered by respondent. Specification is alternative-specific MNL (ASCL) with attributes common across alternatives and ASCs for “Device A” and “Device B” (No Device is the reference). Joint test of feedback shifts on ASCs:* $\chi^{2}(2)=9.46, p=0.0088$*. Model 1 diagnostics: log pseudolikelihood = −4747.040;* $Wald \chi^{2}(10)=570.47 (p<0.001).$ *Model 2 diagnostics: log pseudolikelihood = −2422.798;* $Wald \chi^{2}\left( 12 \right)=268.35 \left( p<0.001 \right).$ *Predicted* $Pr(No device): feedback - nofeedback = +0.0846$ *(cluster-bootstrap 95% CI 0.0832 - 0.0859).*

**Figure S1. Predicted probability of choosing “No device” by feedback status**

**Supplementary Table S2. Site heterogeneity (Duke vs national panel): pooled interactions and stratified models**

*(feedback-eligible subsample; robust SEs clustered by respondent)*

**Panel A. Pooled conditional logit with site interactions (base=Panel; Duke coded 1)**

| **Parameter (reference in parentheses)** | **Coef.** | **SE** | **z** | **p-value** |
| --- | --- | --- | --- | --- |
| **Attributes (Panel base)** |  |  |  |  |
| 1-year gain in NYHA II | 0.657 | 0.084 | 7.80 | <0.01 |
| 1-year gain in NYHA III | 0.509 | 0.079 | 6.42 | <0.01 |
| **30-day mortality (vs 0%)** |  |  |  |  |
| 2% | -0.222 | 0.068 | -3.26 | 0.01 |
| 5% | -0.569 | 0.091 | -6.24 | <0.01 |
| 10% | -1.084 | 0.145 | -7.50 | <0.01 |
| 15% | -1.193 | 0.133 | -8.96 | <0.01 |
| **In-hospital complications (vs 0%)** |  |  |  |  |
| 5% | -0.191 | 0.073 | -2.63 | 0.09 |
| 15% | -0.580 | 0.090 | -6.41 | <0.01 |
| 40% | -0.966 | 0.109 | -8.87 | <0.01 |
| Remote device adjustment: Yes (vs No) | 0.165 | 0.060 | 2.72 | 0.06 |
| Opt-out (No Device) | -1.499 | 0.230 | -6.52 | <0.01 |
| Feedback x Opt-out | 0.799 | 0.267 | 2.99 | 0.03 |
| **Duke interactions (difference from Panel)** |  |  |  |  |
| Duke x NYHA II | 0.430 | 0.191 | 2.25 | 0.024 |
| Duke x NYHA III | 0.168 | 0.173 | 0.97 | 0.333 |
| **30-day mortality** |  |  |  |  |
| Duke x 2% | 0.014 | 0.148 | 0.10 | 0.924 |
| Duke x 5% | 0.159 | 0.174 | 0.91 | 0.362 |
| Duke x 10% | 0.047 | 0.345 | 0.14 | 0.892 |
| Duke x 15% | -0.083 | 0.307 | -0.27 | 0.787 |
| **Complications** |  |  |  |  |
| Duke x 5% | 0.009 | 0.183 | 0.05 | 0.961 |
| Duke x 15% | 0.163 | 0.192 | 0.85 | 0.395 |
| Duke x 40% | 0.088 | 0.203 | 0.43 | 0.664 |
| Duke x Remote adjustment | -0.068 | 0.139 | -0.49 | 0.624 |
| Duke x Opt-out | 0.302 | 0.490 | 0.62 | 0.538 |
| Duke x Feedback x Opt-out | -0.353 | 0.619 | -0.57 | 0.568 |
| **Model information** |  |  |  |  |
| Observations | 8,160 |  |  |  |
| Respondents | 340 |  |  |  |
| Log pseudolikelihood | -2,417.626 |  |  |  |
| Wald $\chi^{2}$ (df) | 385.78 (24), p<0.01 |  |  |  |
| Joint test: all Duke interactions | χ²(11) =8.10, p=0.705 |  |  |  |
| Test: Duke difference in Feedback x Opt-out | χ²(1) = 0.33, p=0.568 |  |  |  |

***Notes:*** *Conditional (fixed effects) logit; robust SEs clustered by respondent. “Panel” is the national online cohort (base); “Duke” is the DUHS cohort. Duke x (·) rows report Duke-Panel differences.* $Feedback\times Optout$ *is a case-level interaction.*

**Supplementary Table S3. Site-stratified conditional logit models**

**Panel S3-A. Duke-only conditional logit**

| **Parameter (reference in parentheses)** | **Coef.** | | | **SE** | | **z** | | **p-value** | |
| --- | --- | --- | --- | --- | --- | --- | --- | --- | --- |
| 1-year gain in NYHA II | 1.087 | | | 0.172 | | 6.31 | | <0.01 | |
| 1-year gain in NYHA III | 0.677 | | | 0.155 | | 4.37 | | <0.01 | |
| **30-day mortality (vs 0%)** |  | | |  | |  | |  | |
| 2% | -0.208 | | | 0.133 | | -1.56 | | 0.118 | |
| 5% | -0.410 | | | 0.150 | | -2.74 | | <0.01 | |
| 10% | -1.037 | | | 0.315 | | -3.29 | | <0.01 | |
| 15% | -1.276 | | | 0.278 | | -4.58 | | <0.01 | |
| **Complications (vs 0%)** |  | | |  | |  | |  | |
| 5% | -0.182 | | | 0.169 | | -1.07 | | 0.283 | |
| 15% | -0.417 | | | 0.170 | | -2.45 | | 0.014 | |
| 40% | -0.878 | | | 0.172 | | -5.11 | | <0.01 | |
| Remote device adjustment: Yes (vs No) | 0.096 | | | 0.126 | | 0.76 | | 0.445 | |
| Opt-out (No Device) | -1.197 | | | 0.436 | | -2.75 | | <0.01 | |
| Feedback x Opt-out | 0.446 | | | 0.561 | | 0.79 | | 0.427 | |
| Log pseudolikelihood | -465.064 | | |  | |  | |  | |
| Observations | | 1,680 |  | |  | |  | |  |
| Respondents | | 70 |  | |  | |  | |  |
| Wald $Chi2$ (df) | 100.65 (12), p<0.01 | | |  | |  | |  | |

**Panel S3-B. Panel-only conditional logit**

| **Parameter (reference in parentheses)** | | **Coef.** | | **SE** | | **z** | | **p-value** | |
| --- | --- | --- | --- | --- | --- | --- | --- | --- | --- |
| 1-year gain in NYHA II | | 0.657 | | 0.084 | | 7.80 | | <0.01 | |
| 1-year gain in NYHA III | | 0.509 | | 0.079 | | 6.41 | | <0.01 | |
| **30-day mortality (vs 0%)** | |  | |  | |  | |  | |
| 2% | | -0.222 | | 0.068 | | -3.26 | | <0.01 | |
| 5% | | -0.569 | | 0.091 | | -6.24 | | <0.01 | |
| 10% | | -1.084 | | 0.145 | | -7.50 | | <0.01 | |
| 15% | | -1.193 | | 0.133 | | -8.96 | | <0.01 | |
| **Complications (vs 0%)** | |  | |  | |  | |  | |
| 5% | | -0.191 | | 0.073 | | -2.63 | | <0.01 | |
| 15% | | -0.580 | | 0.090 | | -6.40 | | <0.01 | |
| 40% | | -0.966 | | 0.109 | | -8.87 | | <0.01 | |
| Remote device adjustment: Yes (vs No) | | 0.165 | | 0.061 | | 2.72 | | <0.01 | |
| Opt-out (No Device) | | -1.499 | | 0.230 | | -6.52 | | <0.01 | |
| Feedback x Opt-out | | 0.799 | | 0.267 | | 2.99 | | <0.01 | |
| Observations | 6,480 | |  | |  | |  | |  |
| Respondents | 270 | |  | |  | |  | |  |
| Log pseudolikelihood | | -1,952.562 | |  | |  | |  | |
| Wald $\chi2$ (df) | | 283.75 (12), p < 0.01 | |  | |  | |  | |

***Notes:*** *NYHA=New York Heart Association. Conditional (fixed effects) logit; robust SEs clustered by respondent. “Panel” is the national online cohort (base); “Duke” is the DUHS cohort. Duke x (·) rows report Duke-Panel differences.* $Feedback\times Optout$ *is a case-level interaction (feedback active at the choice set). Whether to pool the two sources of recruitment was assessed via joint Wald test of site interactions: difference in feedback effect assessed via Wald test of* $Duke\times Feedback\times Optout$*.*

**Supplementary Table S4. Model-based site contrasts for the feedback effect on opting out**

| **Contrast / Test** | **Estimate** | **SE** | $\boldsymbol{z / \chi^{2}}$ | **p-value** | **Interpretation** |
| --- | --- | --- | --- | --- | --- |
| Pooled Feedback x Optout (utility scale) | 0.799 | 0.267 | z=2.99 | <0.01 | Feedback increases utility of opting out overall. |
| Duke-only Feedback x Optout | 0.446 | 0.561 | z=0.79 | 0.427 | Positive but imprecise in smaller Duke sample. |
| Panel-only Feedback x Optout | 0.799 | 0.267 | z=2.99 | 0.003 | Significant in national panel. |
| Duke difference in Feedback x Optout (pooled) | -0.353 | 0.619 | z=-0.57 | 0.568 | No evidence that the feedback effect differs by site. |
| Joint test: any Duke taste heterogeneity (pooled) | - | - | $\chi^{2}$ (11)=8.10 | 0.705 | No systematic site taste heterogeneity. |

***Note:*** *Estimates are on the utility (logit) scale from the pooled or stratified conditional logit models. See Supplementary Tables S1-S2 for full coefficient sets.*

**Table S5. Multinomial logit (preference-space) with extended scale covariates: utility and scale parameters**

| **Panel A. Utility coefficients (choice model)** | **Coef.** | **SE** | **z-score** | **p-value** |
| --- | --- | --- | --- | --- |
| **Opt-out (No Device) vs Device** | -0.753 | 0.266 | -2.83 | 0.01 |
| **Device ASC (alternative-specific constant)** | 0.153 | 0.048 | 3.18 | <0.01 |
| **Physical functioning** |  |  |  |  |
| 1-year gain to NYHA class II (vs none) | 0.618 | 0.076 | 8.15 | <0.01 |
| 1-year gain to NYHA class III (vs none) | 0.435 | 0.068 | 6.44 | <0.01 |
| **30-day mortality risk (vs 0%)** |  |  |  |  |
| 2% | -0.147 | 0.064 | -2.27 | 0.023 |
| 5% | -0.433 | 0.075 | -5.81 | <0.01 |
| 10% | -0.981 | 0.116 | -8.43 | <0.01 |
| 15% | -1.028 | 0.121 | -8.50 | <0.01 |
| **In-hospital complication risk (vs 0%)** |  |  |  |  |
| 5% | -0.126 | 0.064 | -1.97 | 0.049 |
| 15% | -0.452 | 0.075 | -6.01 | <0.01 |
| 40% | -0.841 | 0.094 | -8.98 | <0.01 |
| **Remote device adjustment: Yes (vs No)** | 0.159 | 0.042 | 3.78 | <0.01 |
| **Opt-out x Feedback** | 0.344 | 0.154 | 2.24 | 0.025 |
| **Opt-out x respondent characteristics** |  |  |  |  |
| Female | 0.172 | 0.133 | 1.29 | 0.197 |
| Older | 0.267 | 0.167 | 1.60 | 0.109 |
| Married | 0.012 | 0.128 | 0.09 | 0.927 |
| High-school or less | 0.240 | 0.163 | 1.47 | 0.141 |
| No college | 0.153 | 0.211 | 0.73 | 0.468 |
| No degree | -0.342 | 0.200 | -1.71 | 0.087 |
| Graduate degree | -0.251 | 0.219 | -1.15 | 0.252 |
| Known device type | 0.239 | 0.129 | 1.85 | 0.064 |
| Prior heart attack | -0.201 | 0.141 | -1.43 | 0.154 |
| White | -0.522 | 0.141 | -3.70 | <0.01 |
| Retired | 0.301 | 0.166 | 1.81 | 0.069 |
| Prior surgery | 0.2562 | 0.161 | 1.59 | 0.111 |
| Disabled | 0.129 | 0.148 | 0.88 | 0.381 |
| NYHA I/II symptoms | -0.662 | 0.149 | -4.45 | <0.01 |
| Arrhythmia | -0.277 | 0.193 | -1.44 | 0.151 |
| Previous device implanted | -0.551 | 0.167 | -3.31 | <0.01 |

| **Panel B. Scale (error variance) covariates** | | **Coef.** | **SE** | **z-score** | | **p-value** |  |
| --- | --- | --- | --- | --- | --- | --- | --- |
| Feedback in scale | | -0.284 | 0.108 | -2.63 | | 0.01 |  |
| At least 8 comprehension questions correct | | 0.291 | 0.096 | 3.04 | | <0.01 |  |
| At least 9 comprehension questions correct | | 0.283 | 0.129 | 2.19 | | 0.028 |  |
| Response time (Fast completion) | | -0.279 | 0.107 | -2.61 | | 0.01 |  |
| Response time (Slow completion) | | 0.070 | 0.091 | 0.78 | | 0.437 |  |
| DUHS site | | 0.215 | 0.095 | 2.26 | | 0.024 |  |
| Confidence filling out medical forms | | -0.631 | 0.211 | -2.99 | | <0.01 |  |
| Very certain about the choices made (vs not certain) | | 0.306 | 0.086 | 3.54 | | <0.01 |  |
| Very easy to answer the choice questions (vs no) | | 0.119 | 0.123 | 0.98 | | 0.328 |  |
| **Model diagnostics** | |  |  |  | |  |  |
| LL (convergence) | -2346.84 |  | | |  | | |
| McFadden’s pseudo-R² | 0.125 |  | | |  | | |
| AIC/n | 1.754 |  | | |  | | |
| BIC/n | 1.836 |  | | |  | | |
| Number of choices | 2,720 |  | | |  | | |
| Number of respondents | 340 |  | | |  | | |
| Number of parameters | 38 |  | | |  | | |
